# Bioassay-Guided Interpretation of Antimicrobial Compounds in Kumu, a TCM Preparation From *Picrasma quassioides’* Stem via UHPLC-Orbitrap-Ion Trap Mass Spectrometry Combined With Fragmentation and Retention Time Calculation

**DOI:** 10.3389/fphar.2021.761751

**Published:** 2021-10-27

**Authors:** Haibo Hu, Changling Hu, Jinnian Peng, Alokesh Kumar Ghosh, Ajmal Khan, Dan Sun, Walter Luyten

**Affiliations:** ^1^ Department of Biology, Animal Physiology and Neurobiology Section, KU Leuven, Leuven, Belgium; ^2^ National Engineering Research Center for Modernization of Traditional Chinese Medicine - Hakka Medical Resources Branch, School of Pharmacy, Gannan Medical University, Ganzhou, China; ^3^ Laboratory for Functional Foods and Human Health, Center for Excellence in Postharvest Technologies, North Carolina Agricultural and Technical State University, North Carolina Research Campus, Kannapolis, NC, United States; ^4^ College of Life Sciences, NanKai University, Tianjin, China

**Keywords:** *Picrasma quassioides*, kumu, beta-carboline, orbitrap elite, MS Fragmenter, fragmentation prediction

## Abstract

The stem of *Picrasma quassioides* (PQ) was recorded as a prominent traditional Chinese medicine, Kumu, which was effective for microbial infection, inflammation, fever, and dysentery, etc. At present, Kumu is widely used in China to develop different medicines, even as injection (Kumu zhusheye), for combating infections. However, the chemical basis of its antimicrobial activity has still not been elucidated. To examine the active chemicals, its stem was extracted to perform bioassay-guided purification against *Staphylococcus aureus* and *Escherichia coli*. In this study, two types of columns (normal and reverse-phase) were used for speedy bioassay-guided isolation from Kumu, and the active peaks were collected and identified via an UHPLC-Orbitrap-Ion Trap Mass Spectrometer, combined with MS Fragmenter and ChromGenius. For identification, the COCONUT Database (largest database of natural products) and a manually built PQ database were used, in combination with prediction and calculation of mass fragmentation and retention time to better infer their structures, especially for isomers. Moreover, three standards were analyzed under different conditions for developing and validating the MS method. A total of 25 active compounds were identified, including 24 alkaloids and 1 triterpenoid against *S. aureus*, whereas only β-carboline-1-carboxylic acid and picrasidine S were active against *E. coli*. Here, the good antimicrobial activity of 18 chemicals was reported for the first time. Furthermore, the spectrum of three abundant β-carbolines was assessed via their IC_50_ and MBC against various human pathogens. All of them exhibited strong antimicrobial activities with good potential to be developed as antibiotics. This study clearly showed the antimicrobial chemical basis of Kumu, and the results demonstrated that HRMS coupled with MS Fragmenter and ChromGenius was a powerful tool for compound analysis, which can be used for other complex samples. Beta-carbolines reported here are important lead compounds in antibiotic discovery.

## Introduction

*Picrasma quassioides* (D. Don) Benn, a prominent medicinal herb from southern and eastern Asia, named “Kumu,” “Kudanmu,” “Shanxiongdan,” and “Kupizi” in China due to its extremely bitter and lasting taste, is a deciduous tree of the Simaroubaceae family. It was described with a scientific name in 1884 (Flora of China Editorial Committee, 1997; Flora of China Editorial Committee, 2008), but as a medicine, its stem in slices or powder was first recorded as “Shanxiongdan” in Xin Yi Xue (AD 1972) for the treatment of dysentery, infections of the biliary tract, and burns and wounds (Medical Team of, 1972; Chinese Materia Medica Editoral Committee of National Administration of TCM, 1999). Although PQ does not have as long a documented history as other TCMs (traditional Chinese medicines), it has received much attention due to its effectiveness in inflammation, infection, and cancer. By now, PQ is widely used in China in different medicinal products, such as Kumu Xiaosanpian (tablet), Fufang Kumu Xiaoyanjiaonang (capsule), Kumu Zhusheye (injection) for treating influenza, upper respiratory tract infections, acute tonsillitis, enteritis, and bacterial dysentery. According to the Chinese Pharmacopeia, its dried stem and leaf are used as Kumu for anti-inflammation, microbial infection, fever, dysentery, and snake or insect bites. However, PQ’s thick stems are commonly used in TCM markets. Also, the Tamang people exploit its wood for fever and joint pain in Nepal (Ambu et al., 2020). In Korea and Japan, the heartwood of PQ, named ‘Picrasma wood’, is used as a herbal drug, consisting of chips, slices, or short pieces of wood (Ministry of Food and Drug Safety, 2012; The ministry of health and labour and welfare, 2016).

Phytochemical studies showed that alkaloids (β-carbolines, canthinones, and cinnamamides), triterpenoids (quassinoids, apotirucallanes, tirucallanes, and apotirucallanes), neolignans, and flavonoids are the major compounds in PQ (Jiao et al., 2010a; Xu et al., 2016; Bai et al., 2020; Mohd Jamil et al., 2020; Ren et al., 2020). They exhibit antitumor (Xie et al., 2020), anti-inflammatory (Jiao et al., 2011), antimicrobial (Chen et al., 2009b; Zhang J. et al., 2019), antiparasitic (Curcino Vieira and Braz-Felho, 2006), anti-dyspepsia (Babu Rao et al., 2013), antihypertensive (Zhao et al., 2013), anti-asthma (Shin et al., 2014), antioxidant (Jung et al., 2012), anti-osteoporosis (Kong et al., 2017), neuroprotective (Zhao et al., 2019c; Guo et al., 2019), and other biological activities. PQ also showed some toxicity and adverse effects in zebrafish embryos (Gong et al., 2018), cultured cells (Lou et al., 2018), and in clinical studies (Weiqi et al., 2019). However, comprehensive studies investigating PQ’s antimicrobial chemicals are still scarce, and the NMR chemical identification usually required milligrams of pure compound, which is time-consuming to purify. In recent years, the advanced Orbitrap Elite Hybrid Mass Spectrometer, which combines a novel high-field orbitrap mass analyzer with a premier dual-pressure linear ion trap mass spectrometer, greatly improved MS and MS^n^ performance and versatility, and it has become a reliable technique for a targeted and a non-targeted analysis of the chemical structure, requiring only picogram of the sample (Denisov et al., 2012; Jiang et al., 2020; Zhao et al., 2020). LC-HRMS/MS (high-resolution mass spectrometry) and MS^n^ provide retention time, extract *m/z* and its fragmentation, from which the structure normally can be inferred based on comparison with standards, or published spectra of compound databases, MS/MS libraries, and molecular networking (Allen et al., 2014; Schymanski et al., 2014; Fisher et al., 2021). But, it is still a challenge to distinguish isomers with similar structures. Recently, several computer-assisted structure elucidation software packages for MS were developed offering new ways for isomer interpretation, such as MS fragmentation prediction (MFP) (Tyrkkö et al., 2010; Hu et al., 2021; Krettler and Thallinger, 2021), retention time calculation (RTC) (Noreldeen et al., 2018; Aalizadeh et al., 2019), and ion mobility spectrometry tools (IMS)(Campuzano et al., 2012; Dodds et al., 2017). With contrast to MFP and RTC, IMS measures the collisional cross section of compounds based on their time-of-flight via a buffer gas in a drift tube, which is not available for all instruments, including orbitraps. Hence, in this study, MFP and RTC (MS Fragmenter and ChromGenius from ACD/Structure Elucidator Suite) were used to perform fragmentation and retention time calculations, aiding for the MS elucidation.

To figure out the active compounds in Kumu, two types of columns (normal and reverse phases) were utilized for fast separation of the more widely used PQ stem. Based on the bioassay test, the active peaks were collected and detected by LC-HRMS/MS for MS^n^. Meanwhile, a compound database of PQ was manually built for MS interpretation, and chemical structures were elucidated based on MS^n^ ion fragments and retention time, comparing with several standards and the database. Notably, for “unknown” compounds (lack of authentic standards and scarce information, such as spectra or fragmentation), the fragmentation prediction program (MS Fragmenter) and the retention time calculation program (ChromGenius) were used to evaluate the chemical structures via predicting mass spectral fragments based on the cleavage rules, and calculating their retention time based on chemical similarity searching. Then all the active compounds were identified. This study demonstrates a rapid, reliable method to isolate and identify natural products via columns, LC-HRMS/MS, and computer-assisted interpretation softwares. The results will provide a basis for further development and utilization of Kumu.

## Materials and Methods

### Preparation of Reagents and Materials

For MS samples, three standard compounds were used in this study: methylnigakinone, nigakinone, and β-carboline-1-carboxylic acid. The first two were previously isolated and identified in our lab via MS and NMR comparison (Gong et al., 2016), and β-carboline-1-carboxylic acid (LOT: 2015-0005238) was purchased from Enamine Ltd (Kyiv, Ukraine). The purity of all chemicals was over 95%. Water was generated by a Milli-Q system (Millipore, Bedford, MA, United States), and formic acid, acetonitrile, methanol, and ammonium acetate (LC/MS grade) were from Thermo Fisher Scientific (Fair Lawn, NJ, United States). The stock solutions of three standards in methanol were prepared at 4 μg/ml and an additional mixed solution for MS detection, stored at 4°C until use.

**FIGURE 1:**
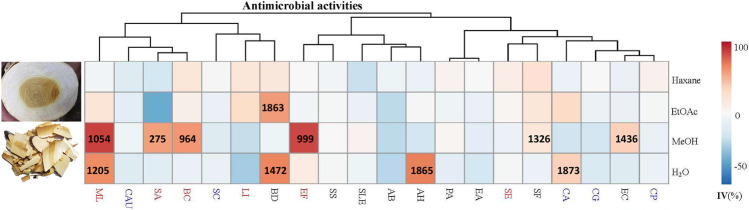
Antimicrobial activities (IC_50_) (μg/ml) of different solvent extracts of *Picrasma quassioides* stem; IV: inhibition value; The abbreviations are the initials of the scientific names of the tested microorganism, including five fungi [*Candida albicans* (CA), *Candida parapsilosis* (CP), *Candida auris* (CAU), *Candida glabrata* (CG), and *Saccharomyces cerevisiae* (SC)], nine G^–^ bacteria [*Escherichia coli* (EC), *Pseudomonas aeruginosa* (PA), *Shigella sonnei* (SS), *Acinetobacter baumannii* (AB), *Enterobacter aerogenes* (EA), *Brevundimonas diminuta* (BD), *Shigella flexneri* (SF), *Salmonella enterica* subsp. enterica (SLE), and *Aeromonas hydrophila* (AH)], and six G^+^ bacteria [*Staphylococcus aureus* (SA), *Staphylococcus epidermidis* (SE), *Micrococcus luteus* (ML), *Listeria innocua* (LI), *Enterococcus faecalis* (EF), and *Bacillus cereus* (BC)], marked in blue, black, and red, respectively.

For samples of antimicrobial tests, the plant materials were collected from Jinpen Mountain, Xinfeng County, Ganzhou, China (Figure 1). According to Flora of China, the samples were identified as *Picrasma quassioides* (D. Don) Benn. by Professor Haibo Hu. The voucher specimen was kept in our lab at Gannan Medical University (No. PQ-XF1701). PQ stems (Kumu) were dried at 40°C, and then grounded into a fine powder. One gram of each powder was extracted in 10 ml of H_2_O, MeOH, EtOAC, and hexane with the help of sonication (4 × 15  min with 4–6  h gap). Then after 5  min centrifuging at 3500 rpm, a 1-ml aliquot of each supernatant was sampled and evaporated in a fume hood (hexane and ethyl acetate), or dried (methanol and water) in a SpeedVac concentrator (SVC 200 H, Stratech Scientific, London, United Kingdom). The dried extracts and three standards were redissolved in DMSO as a 20 mg/ml for antimicrobial assessment. All the samples were stored at 4°C for further testing.

### Antimicrobial Assay

The antibacterial activity of crude extracts was tested against 20 human pathogenic strains, including five fungi [*Candida albicans* (SC 5314), *Candida parapsilosis* (ATCC 22019), *Candida auris* (OS299), *Candida glabrata* (ATCC 2001), and *Saccharomyces cerevisiae* (ATCC 7754)], six Gram-positive bacteria [*Staphylococcus aureus* (ATCC6538, Rosenbach), *Staphylococcus epidermidis* (ATCC 1457), *Micrococcus luteus* (DPMB 3), *Listeria innocua* (LMG 11387), *Enterococcus faecalis* (HC-1909-5), and *Bacillus cereus* (LMG9610)], and nine Gram-negative bacteria [*Escherichia coli* (ATCC 47076), *Pseudomonas aeruginosa* (PAO1), *Shigella sonnei* (LMG 10473), *Acinetobacter baumannii* (RUH134), *Enterobacter aerogenes* (or *Klebsiella aerogenes*, ATCC 13048), *Brevundimonas diminuta* (a gift from Prof. Rob Lavigne at KU Leuven), *Shigella flexneri* (LMG10472), *Salmonella enterica* subsp. enterica (ATCC 13076), and *Aeromonas hydrophila* (ATCC 7966)]. The antimicrobial tests were performed by a 96-well microdilution method. In brief, for bacteria and fungi, 10 and 5 μl, respectively, of a two-fold dilution series of each extract (20 mg/ml stock), blank controls (DMSO) and positive controls (ciprofloxacin, 100 μg/ml stock and miconazole, 200 μg/ml stock) were transferred into 96-well plates with the test microogranism (OD: 0.003) and cultured for 20 h. Then the inhibition value (%, IV) was obtained by dividing the sample’s OD value minus that of an extract control without inoculation by the average OD of the blank controls (solvent) and multiplying by 100.

All the tests were repeated for confirmation, and IC_50_ (minimum inhibitory concentration to inhibit the growth of 50%) was calculated by dose-response fitting using non-linear least-squares sigmoid regression (GraphPad Prism 7.0, San Diego, CA). The data were clustered using a webtool (https://biit.cs.ut.ee/clustvis/) to obtain clustered heatmaps. MBC (minimum bactericidal concentration) was obtained directly from the agar tests of serial chemical dilution.

### Fractionation and Isolation of Constituents

100 g of PQ stem was extracted three times aided by sonication (4 × 30 min with 4–6  h gap) with the selected solvent (methanol) according to the antimicrobial activities, and then dried in a Rotavapor (R-100, BÜCHI, Flawil, Switzerland). Then, 5.6 g of extract was finally obtained and mixed with 25 g silica gel (60 Å, LOT#MKCK 1888; Sigma-Aldrich, Germany). Afterward, a large-scale separation was performed on a silica gel column (ECOPLUS 35 × 500 mm, YMC, Kyoto, Japan) in a preparative liquid chromatography setup (Waters Delta 600 multi-solvent quaternary pump, Waters detector 2487, Waters 600 controller, Massachusetts, United States) using a mobile phase consisting of hexane (A), ethyl acetate (B), methanol (C), and 25% acetic acid in methanol (D), performed a flow rate of 40 ml/min, with a step gradient from 95% A and 5% B to 100% D (5–20% step every 10 min). Finally, 218 fractions were collected (1 fraction per minute) and 1 ml of each fraction was sampled and dried; then 100 μl DMSO was added per sample to prepare stocks for antimicrobial testing.

Based on the bioactivity results of 218 fractions, seven active fractions (F47, F60, F88, F108, F118, F125, and F181) were selected for further separation via HPLC (LC-20AT pumps combined with a SPD-M20A detector and DGU-20A_3R_ degasser, SHIMADZU, Kyoto, Japan). Separation conditions of each fraction were optimized on several analytical columns (250 × 4.6 mm, 5 μm, C18, Symmetry, Waters; 150 × 4.6 mm, 4 μm, Polar-RP, Synergi, Phenomenex; 250 × 4.6 mm, 4 μm, Hydro-RP, Synergi, Phenomenex), and then were geometrically transferred to semi-preparative columns to ensure the same selectivity for peak collections. F47 and F60 were separated on a semi-preparative C18 column (250 × 10 mm, 5 μm, C18, Sunfire, Waters) and the rest by a Polar-RP column (250 × 10 mm, 4 μm, Polar-RP, Synergi, Phenomenex) using a mobile phase consisting of water (A) and acetonitrile (B), both containing 0.1% formic acid. For each fraction, the injected amount was kept around 2 mg, and all the subfraction (1 per minute) were collected via gradient elution with a 4 ml/min flow rate using the conditions listed in Table 1. After drying each fraction in a SpeedVac, 15 μl DMSO were added to each tube for antimicrobial testing. To eliminate possible errors during a single run, all subfractions were collected and tested twice. This was also done for all the active peaks collected under the same condition to confirm the activities. Then the confirmed peaks were collected, dried, and kept in 4°C for the MS analysis.

**TABLE 1:** The HPLC gradient conditions of the selected fractions.

Fractions	Gradients
F47	0–8 min, 50% B; 8–65 min, 50–100% B; 65–80 min, 100% B
F60	0–8 min, 25% B; 8–60 min, 25–50% B; 60–70 min, 50–100% B; 70–80 min, 100% B
F88	0–5 min, 15% B; 5–55 min, 15–30% B; 55–65 min, 30–50% B; 65–70 min, 30–100% B; 70–80 min, 100% B
F108	0–8 min, 10% B; 8–15 min, 10–30% B; 15–65 min, 30–55% B; 65–70 min, 55–100% B; 70–80 min, 100% B
F118	0–8 min, 10% B; 8–15 min, 10–30% B; 15–65 min, 30–65% B; 65–70 min, 65–100% B; 70–80 min, 100% B
F125	0–8 min, 10% B; 8–60 min, 10–60% B; 60–70 min, 60–100% B; 70–80 min, 100% B
F181	0–5 min, 10% B; 5–10 min, 10–20% B; 10–63 min, 20–60% B; 63–70 min, 60–100% B; 70–80 min, 100% B

### UHPLC and MS/MS Conditions

The UHPLC-MS/MS analysis was performed with an Ultimate 3000 UHPLC system (Dionex Thermo Scientific) equipped with an EasySpray C18 column (2 μm, 100 Å, 50 μm × 15 cm, Thermo Scientific), and Orbitrap Elite High-Field Orbitrap Hybrid Mass Spectrometer (Thermo Scientific, United States), which consisted of orbitrap high-resolution selection and ion trap scanning, was used for the chemical analysis of natural small-molecular compounds. With a 0.3 μl·min^−1^ flow rate, the C18 column was set at 35°C and the injection volume was 5 μl. The mobile phases were composed of water (eluent A) and 80% acetonitrile with 20% water (eluent B), both containing 0.1% formic acid (for negative mode detection, 0.1% ammonium acetate was used instead of formic acid). The gradients were set as follows: 0–3 min, 5% B; 3–20 min, 5%–100% B; 20–23 min, 100% B; 23–24 min, 100%–5% B; and 24–34 min, 5% B to re-equilibrate the system.

For MS detection, the Orbitrap-Ion Trap mass spectrometer was fitted with a heated electrospray ionization (ESI) ion source, and both negative and positive ionization modes were performed at a full scan mode ranging from *m*/*z* 100–2000. To aid the structural identification of the components, top 20 ions’ MS/MS fragmentation (dd-MS^2^-TOP 20) was operated with an *m*/*z* range of 50–2000. The MS parameters were optimized as reported previously (Hu et al., 2021) with the followings: spray voltage −2.1 Kv/+2.1 Kv; capillary temperature, 275°C; multipole RF amplifier, 800 Vp-p; reagent ion source temperature, 160°C. The stepped ITMS (Ion trap mass spectrometry) was set to 35 eV for MS/MS acquisition of the most intense ions from FTMS (Fourier transform mass spectrometry).

### Data Processing and Analysis

All the MS data acquisition and analyses were done by Xcalibur 4.2 (Thermo Scientific, United States), ACD/MS Workbook Suite 2020, combined with MS Fragmenter, and ChromGenius (ACD/labs, Canada). The original UHPLC-MS data of each sample were exported, and their background was subtracted based on MS data of the blank solvent for both positive and negative ions. Then the processed data were imported into ACD/labs to extract peaks and align chromatograms to make the comparison more accurate than manually, including ITA (itellitarget) and IX 2.0 (IntelliXtract), which are algorithms to analyze LC(GC)/MS datasets for targeted and untargeted component detection and deconvolution, respectively. The assignment accuracy was set to 5 ppm for both.

Moreover, the published components of PQ were collected by checking SciFinder, Web of Science, the Dictionary of Natural Products, China National Knowledge Infrastructure (CNKI), and other databases, and then Spectrus DB (ACD/labs) was used to create a manually built compound library, including chemical names, molecular formulas, exact molecular mass, and structures. The MS^1^ and MS^2^ spectra of active peaks were analyzed by comparing the detected formulas and MS fragments with the component library we built and the COCONUT database (Sorokina et al., 2021) (the largest publicly available natural product database), and all the active compounds were targeted and identified. In detail, the spectrum of standards can figure out themselves easily, and those compounds without standards can be elucidated by their ion fragments and retention time (tR) according to fragmentation prediction by MS Fragmenter and retention time calculation by ChromGenius, from which all the fragmentation patterns of each compound in this study were summarized and further verified.

## Results and Discussion

### Antimicrobial Activities of Kumu

Thus far, antimicrobial tests mainly focused on PQ compounds, especially for its alkaloids. A previous study showed that the extract of total alkaloids exhibited antimicrobial activities against *Streptococcus hemolytic*-β, *Staphylococcus aureus*, *Shigella castellani*, *Bacillus subtilis* subsp, and *Sporosarcina* (Meng, 2007). Total alkaloids were reported to exhibit a wider spectrum and better antimicrobial activity than single alkaloids, possibly due to synergistic effects (Guihua, 2011). Notably, the fat-soluble alkaloids provided good inhibition against both wild type and two highly virulent strains of *E. coli*, even higher than berberine sulfate, mequindox, and ofloxacin, while water-soluble alkaloids hardly had bacteriostatic effects on these strains (He et al., 2008). Also, the essential oil of PQ inhibited both bacterial (e.g. *Bacillus subtilis)* and fungal (e.g. *Ganoderma lucidum)* species (Hanif et al., 2010). However, as a TCM, Kumu was always used as a whole extract. Comprehensive studies investigating the bioactivities of its total extracts are still scarce. Hence, in this study, four different solvents (hexane, ethyl acetate, methanol, and water) were used to extract the stem of PQ, and test these extracts against 20 different human pathogens. The results show that Kumu extracts can inhibit five G^+^, four G^–^ bacteria, and one fungus (Figure 1), in which Kumu exhibited strongest activity against *Staphylococcus aureus* (IC_50_ = 275 μg/ml). These results support Kumu’s medicinal application as an anti-infection agent. Moreover, the results show that methanol is the best solvent to extract Kumu for its antimicrobial activity, and it was used to perform the large-scale extracting for bioassay guided purification work.

### Bioassay-Guided Isolation

Based on the above antimicrobial activities, it is worth performing bioassay-guided purification for identifying the antimicrobial compounds of Kumu. By now, bioassay-guided isolation of natural products have been widely used for different activities, such as anti-inflammatory (Erdemoglu et al., 2008), antioxidant (Yang et al., 2020), anti-seizure (Brillatz et al., 2020b) (Brillatz et al., 2020a), vasorelaxant (Othman et al., 2006), aldose reductase inhibitory (Logendra et al., 2006), anti-plasmodial (Baldé et al., 2021), antiviral (Parvez et al., 2020), antibacterial (He et al., 2017), and antifungal (Yan et al., 2020). But most of these studies were performed with multiple purification steps, which consumed long time and large amounts of solvents. With the rapid development of chromatographic techniques, many different columns have been already used for fast collection of small-amount compounds. Hence, in this study, two types of columns were selected for the separation: a large normal-phase silica gel column and semi-preprative reverse-phase columns. The aim was to rapidly determine and collect the active fractions/peaks to increase the purification efficiency.

**FIGURE 2:**
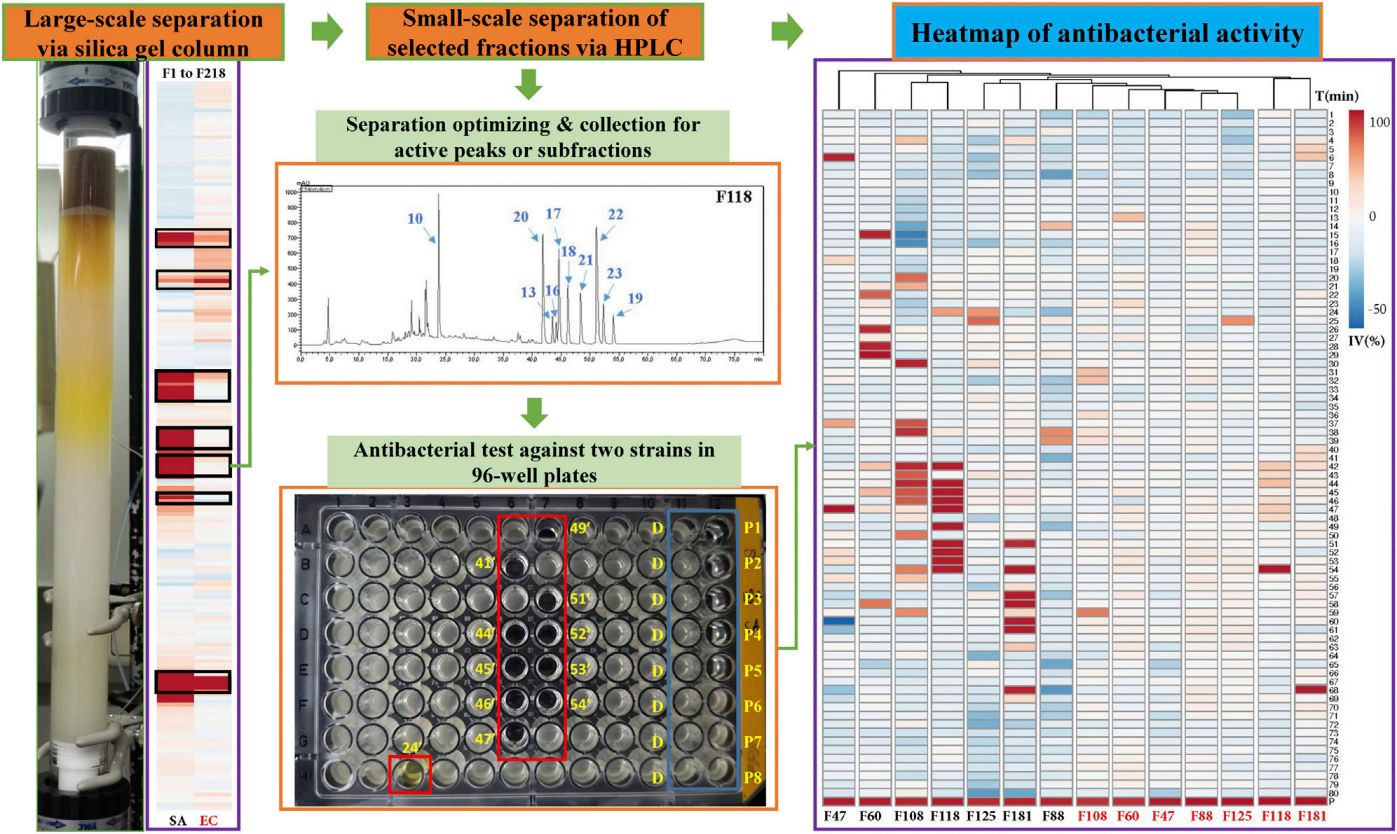
Separation of antibacterial compounds from Kumu based on successive chromatographic columns (normal and reversed-phase chromatography) and the heatmap of the bioactivity of their factions against a representative Gram-positive strain (SA: *S. aureus*) and Gram-negative strain (EC: *E. coli*). F: fractions of preparative silica gel column, D: negative control, DMSO, P1-8: positive control (ciprofloxacin) in different concentrations, IV: inhibition value, T: time for collection in minutes, such as 24’ and 41’.

**FIGURE 3:**
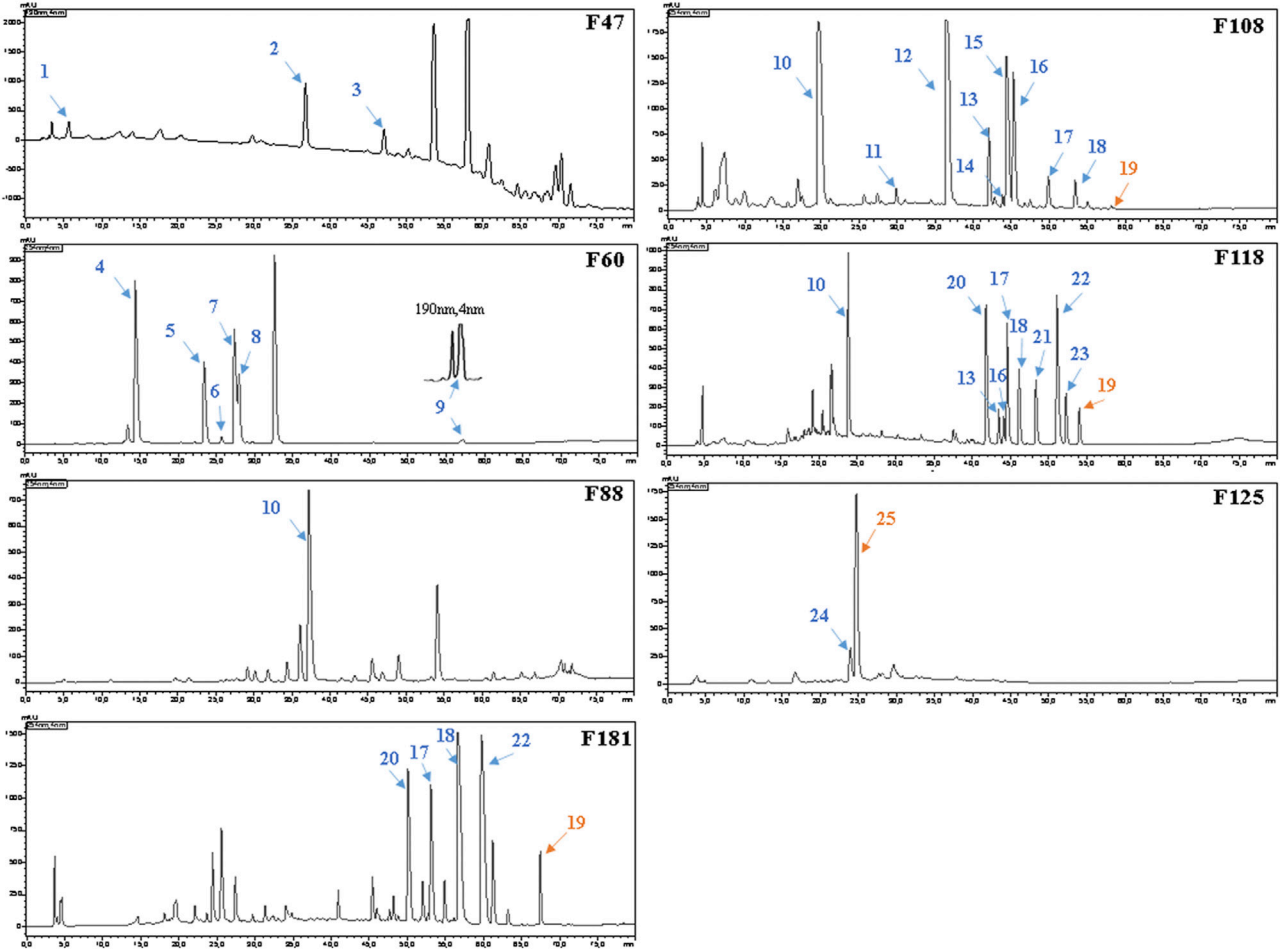
HPLC–UV chromatograms and active peaks of the selected fractions from the stem of *Picrasma quassioides*. The peaks active against *S. aureus* are marked in blue, while the ones active against both *S. aureus* and *E. coli* are numbered in orange.

For the bioassays, *Staphylococcus aureus* (Gram-positive) and *Escherichia coli* (Gram-negative), which belong to top dangerous superbugs worldwide (World Health Organization, 2017), were selected for the antimicrobial activities of Kumu. After separation on a preparative silica gel column, 218 fractions (F1-218) were obtained for antimicrobial testing, and seven active groups were found including F46-52, F54-60, F88-96, F105-112, F113-119, F122-129, and F177-187 (Figure 2). From each, a representative fraction was selected for further purification based on their inhibition values: F47, F60, F88, F108, F118, F125, and F181. By comparing different columns and separation conditions, F47 and F60 were further separated on a semi-preparative C18 column, and the remaining fractions by the Polar-RP column. In either case, we used a mobile phase consisting of water (A) and acetonitrile (B), both containing 0.1% formic acid. Afterward, each HPLC-subfraction was tested against the two aforementioned bacteria, and their activities are shown as a heatmap (Figure 2). The subfractions showed much less activity against *E. coli* than *S. aureus*, presumably because G^–^ bacteria have a multilayer outer membrane preventing many xenobiotics passing through the cell membrane, so it is more difficult to find activity against G^–^ (Randall et al., 2013). Finally, 3, 5, 1, 10, 10, 2, and 5 active peaks were obtained from the seven fractions, respectively, and all these 36 peaks were collected for chemical identification via LC-HRMS/MS (Figure 3).

### Identification of Active Compounds in Selected Fractions via LC-HRMS/MS

**FIGURE 4:**
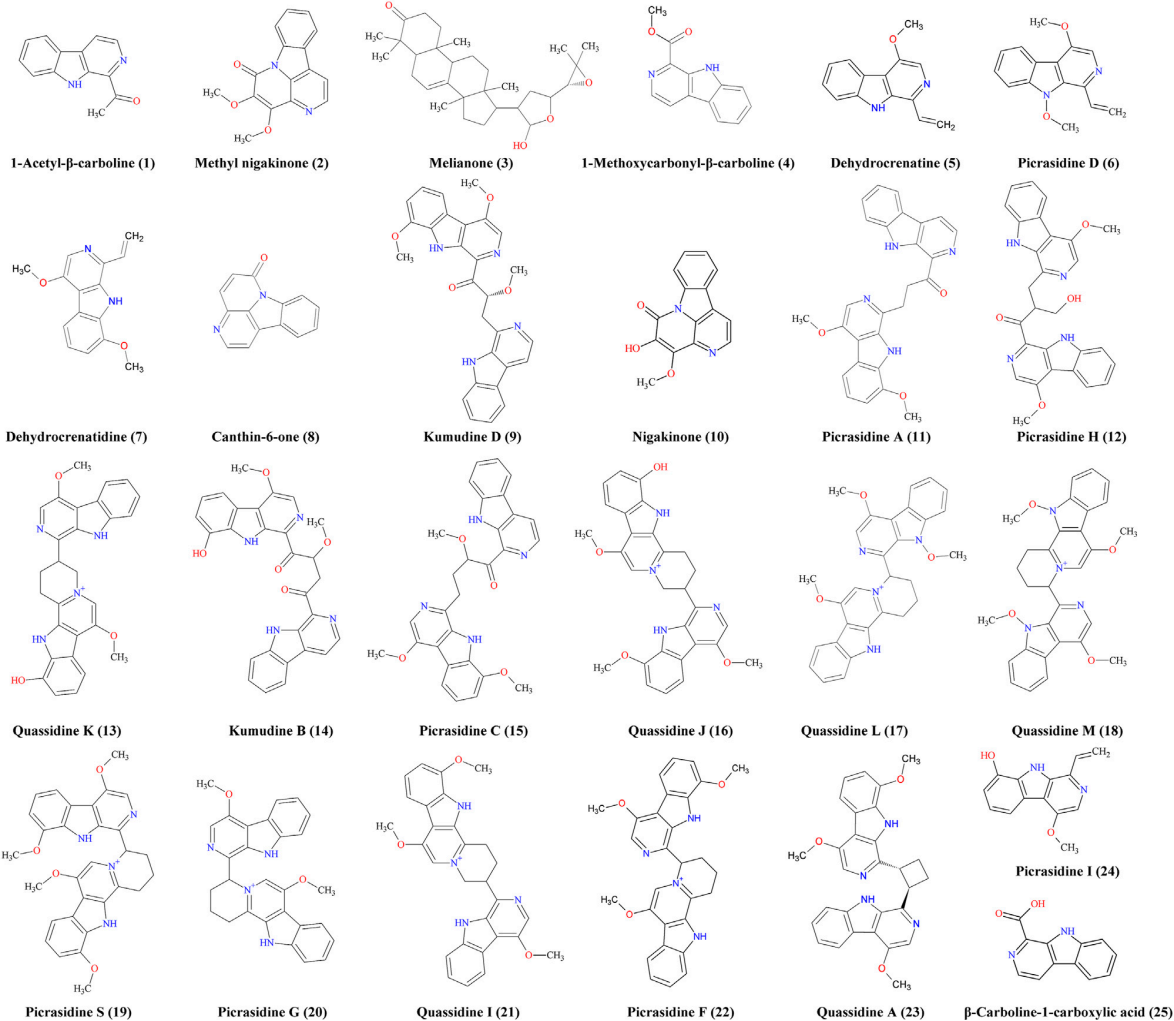
Antimicrobial chemicals identified from the stem of *Picrasma quassioides*.

Before the MS analysis, all peaks were collected and tested to confirm their activities. The results showed that all 36 peaks were active against *S. aureus*, but only four peaks were also active against *E. coli*, including the subfractions at 59, 54, 26 , and 68 min of F108, 118, 125, and 181, respectively. Furthermore, mass responds of pure chemicals were easily affected by various noises of background in total ion chromatograms, due to the high sensitivity of mass detection. Hence, all 36 peaks and several blank samples were analyzed both in positive and negative ion mode by an UHPLC-Orbitrap-Ion-Trap Mass Spectrometer, based on the optimized experimental condition. Then all chemical analyses were confirmed by both positive and negative ions, except in a few cases where data were only available from one ion mode. By comparing with available standards, fragmentation of MS Fragmenter and retention time calculations, a total of 25 active compounds (some peaks were identified as same compounds, in Figure 3) were identified (Table 2), three of which were only detected in positive ion mode under our experiment conditions: 1-acetyl-β-carboline, methylnigakinone, and melianone. All the chemical structures are shown in Figure 4, in which compounds 1–2 and 4–25 are alkaloids, while compound 3 is a triterpenoid. Notably, only picrasidine S (19) and β-carboline-1-carboxylic acid (25) showed strong inhibition against both *S. aureus* and *E. coli*.

**TABLE 2:** Mass information of antimicrobial chemicals identified from the stem of *Picrasma quassioides*.

No	Name	tR (min)	Formula	Ion mode	Calculated *m*/*z*	Observed *m*/*z*	Diff. (ppm)	MS/MS fragments
1	1-Acetyl-β-carboline	18.90	C_13_H_10_N_2_O	[M+H]^+^	211.0866	211.0864	−0.90	211.1406, 194.0908, 193.0662, 185.9508, 169.0260, 166.9848
**2**	**Methylnigakinone**	19.22	C_16_H_12_N_2_O_3_	[M+H]^+^	281.0921	281.0919	−0.60	266.9752, 266.0262, 265.0667, 249.0384, 247.9462, 237.8373, 236.9872, 220.9998, 219.9872
3	Melianone	20.30	C_30_H_46_O_4_	[M+H]^+^	471.3469	471.3472	0.67	453.2476, 441.2062, 435.3094, 425.3902, 399.3133, 395.2563, 381.1285, 313.2863, 221.1231, 218.9906
4	1-Methoxycarbonyl-β-carboline	15.98	C_13_H_10_N_2_O_2_	[M+H]^+^	227.0815	227.0810	−2.22	212.9858, 195.0121, 184.9885, 167.0067
				[M−H]^−^	225.0670	225.0669	−0.23	240.0292, 236.9905, 227.0269, 213.0110, 212.0007
5	Dehydrocrenatine	14.00	C_14_H_12_N_2_O	[M+H]^+^	225.1022	225.1017	−2.40	225.0706, 211.0065, 210.0435, 192.9132, 181.9036
				[M−H]^−^	223.0877	223.0877	0.06	209.1688, 208.1098, 195.1732, 167.6166
6	Picrasidine D	14.33	C_15_H_14_N_2_O_2_	[M+H]^+^	255.1128	255.1129	0.38	255.1180,241.0491, 240.0522, 222.9341
				[M−H]^−^	253.0983	253.0983	0.19	239.0415, 238.1265,223.0002, 118.9094, 88.9246
7	Dehydrocrenatidine	14.45	C_15_H_14_N_2_O_2_	[M+H]^+^	255.1128	255.1126	−0.80	255.0867, 240.9951, 240.0045, 227.0230, 222.8967, 214.0931, 196.1431
				[M−H]^−^	253.0983	253.0983	0.19	245.6254,240.2685,238.1152,223.0610,207.9691
8	Canthin-6-one	18.25	C_14_H_8_N_2_O	[M+H]^+^	221.0709	221.0706	−1.54	221.0535, 193.0897, 166.1287
9	Kumudine D	17.63	C_28_H_24_N_4_O_4_	[M+H]^+^	481.1870	481.1862	−1.73	480.2195, 463.1156, 449.1460, 431.1321, 281.3585, 229.0555, 210.9864
				[M−H]^−^	479.1725	479.1720	−1.00	461.2052, 447.1585, 433.2364, 373.2896, 352.2326
**10**	**Nigakinone**	17.28	C_15_H_10_N_2_O_3_	[M+H]^+^	267.0764	267.0761	−1.19	267.0483, 252.9591, 251.9883, 239.0147, 225.0800, 220.9192, 210.8483
				[M−H]^−^	265.0619	265.0617	−0.62	251.0105, 250.1427, 248.9544, 247.6245, 238.0508, 237.0907, 221.0820
11	Picrasidine A	16.15	C_27_H_22_N_4_O_3_	[M+H]^+^	451.1765	451.1758	−1.48	451.1954, 253.0459, 238.0994, 229.0569, 223.0212, 211.0181, 199.0065
				[M−H]^−^	449.1619	449.1611	−1.81	435.2018, 434.1852, 237.0396, 234.9474, 224.9442, 212.0330, 211.0741, 196.0077
12	Picrasidine H	16.49	C_28_H_25_N_4_O_4_^+^	[M+H]^+^	481.1870	481.1862	−1.73	481.2508, 449.2208, 253.0801, 237.9780, 229.0756, 213.9998
				[M−H]^−^	479.1725	479.1719	−1.21	465.1142, 464.1550, 449.1692, 237.0669, 235.9578, 234.9786, 211.9767, 211.0386
13	Quassidine K	16.92	C_28_H_25_N_4_O_3_^+^	[M]^+^	465.1921	465.1914	−1.54	465.2053, 267.1114, 254.0098, 253.0535, 238.0208, 212.9936, 197.9832
				[M−2H]^−^	463.1776	463.1772	−0.79	449.1972, 448.2293, 433.2408, 265.2134, 237.0870, 235.0272, 221.0503, 195.0514
14	Kumudine B	16.60	C_28_H_22_N_4_O_5_	[M+H]^+^	495.1663	495.1653	−2.01	495.1435, 477.1572, 281.0458, 266.9739, 253.0456, 214.9882
				[M−H]^−^	493.1517	493.1513	−0.90	478.2079, 475.2882, 451.2625, 293.2664, 266.0257, 265.0934, 251.0856, 250.0576, 211.1294
15	Picrasidine C	16.93	C_29_H_26_N_4_O_4_	[M+H]^+^	495.2027	495.2024	−0.57	495.2255, 463.2053, 267.0492, 253.0370, 243.0504, 228.0106
				[M−H]^−^	493.1881	493.1877	−0.87	479.0698, 478.1480, 464.1842, 463.2394, 252.1857, 237.1095, 235.0246, 226.0647, 225.0798, 213.0634, 211.0001
16	Quassidine J	17.02	C_29_H_27_N_4_O_4_^+^	[M]^+^	495.2027	495.2023	−0.77	495.2697, 465.1927, 267.0480, 253.0714, 243.0592, 238.0275, 228.0461, 212.9872
				[M−2H]^−^	493.1881	493.1877	−0.87	478.1840, 364.1169, 251.0574, 234.9872, 225.0534, 211.0231
17	Quassidine L	17.52	C_29_H_27_N_4_O_3_^+^	[M]^+^	479.2078	479.2060	−3.69	479.2004, 464.1393, 449.1678, 447.2045, 267.0383, 255.0499, 237.0422, 227.9599, 212.9119
				[M−2H]^−^	477.1932	477.1929	−0.66	463.2171, 462.2030, 447.1887, 267.1133, 252.0062, 237.0820, 225.0665, 211.0365, 195.0479
18	Quassidine M	17.68	C_30_H_29_N_4_O_4_^+^	[M]^+^	509.2183	509.2172	−2.22	509.2563, 494.1823, 479.1444, 281.0834, 267.0577, 255.0575, 228.0182, 213.8599, 184.9867
				[M−2H]^−^	507.2038	507.2037	−0.15	493.2179, 492.1960, 477.2439, 267.1083, 252.0768, 251.1288, 237.0118, 234.9831, 224.9680, 211.0773
19	Picrasidine S	18.08	C_30_H_29_N_4_O_4_^+^	[M]^+^	509.2183	509.2172	−2.22	509.2216, 479.1555, 478.2160, 295.1020, 266.9344, 255.0566, 242.1013, 227.9459, 194.0148
				[M−2H]^−^	507.2038	507.2038	0.04	492.1746, 477.2206, 252.0334, 251.1116, 235.0350, 225.0618, 211.0239
20	Picrasidine G	17.25	C_28_H_25_N_4_O_2_^+^	[M]^+^	449.1972	449.1967	−1.12	449.2177, 434.1952, 251.0516, 237.1038, 224.9942, 212.9841, 198.0559
				[M−2H]^−^	447.1826	447.1825	−0.33	433.1703, 432.2199, 222.0418, 221.1324, 207.9999, 196.0633, 195.0490, 182.0178
21	Quassidine I	17.02	C_29_H_27_N_4_O_3_^+^	[M]^+^	479.2078	479.2073	−0.97	479.2784, 448.2095, 447.1479, 266.9980, 249.0569, 238.0862, 225.0882, 212.9724, 197.9255
				[M−2H]^−^	477.1932	477.1929	−0.66	463.1853, 462.2612, 447.2323, 445.2561, 251.1134, 225.0477, 221.0837, 210.0430, 195.0394
22	Picrasidine F	17.88	C_29_H_27_N_4_O_3_^+^	[M]^+^	479.2078	479.2072	−1.18	479.1259, 464.1355, 448.1689, 447.1413, 266.9610, 249.1028, 227.9948
				[M−2H]^−^	477.1932	477.1931	−0.24	462.2571, 461.2220, 447.1597, 267.4268, 252.1014, 237.1253, 226.0685, 225.0949, 211.0312, 195.0119
23	Quassidine A	18.08	C_29_H_26_N_4_O_3_	[M+H]^+^	479.2078	479.2069	−1.81	479.2355, 449.1487, 448.1759, 265.0763, 250.9949, 226.9919, 211.0252
				[M−H]^−^	477.1932	477.1935	0.60	462.1905, 447.2411, 267.0988, 252.0888, 237.0792, 225.0339, 211.0342, 195.0640, 182.0502
24	Picrasidine I	12.47	C_14_H_12_N_2_O_2_	[M+H]^+^	241.0972	241.0970	−0.64	241.0725, 223.0385, 221.3182, 194.9022, 180.8792
				[M−H]^−^	239.0826	239.0825	−0.42	221.9767, 221.0481, 196.0063, 195.0665
**25**	**β-Carboline-1-carboxylic acid**	13.88	C_12_H_8_N_2_O_2_	[M+H]^+^	213.0659	213.0660	0.68	212.9659, 195.0410, 166.9938
				[M−H]^−^	211.0513	211.0515	0.94	211.2809, 167.9854, 167.0509, 72.8464

The standards used in this experiment are marked in bold.

### Structure Elucidation Based on Comparison With Published Spectra, Standards, and MS Fragmenter

**FIGURE 5:**
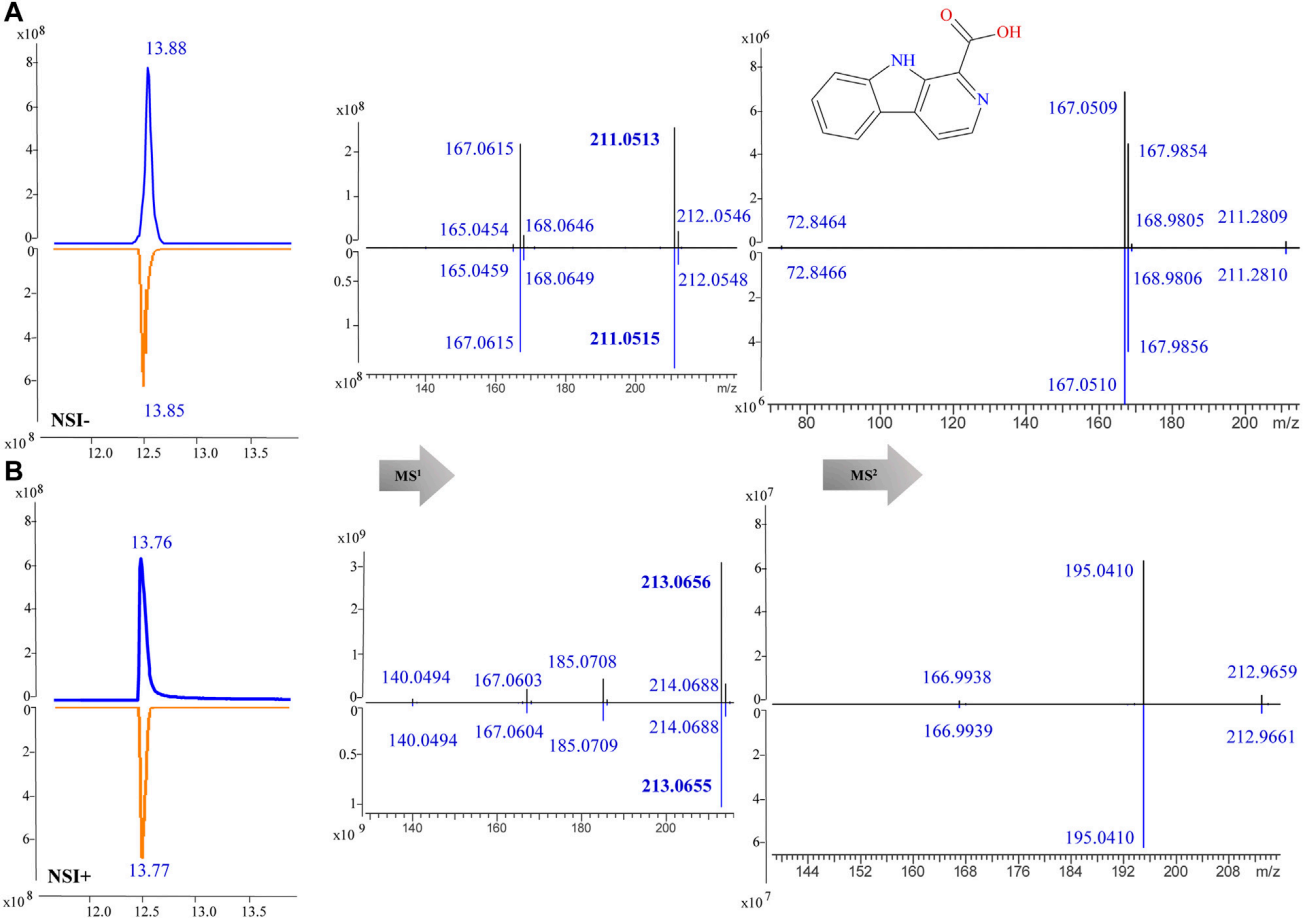
MS^1^ and MS^2^ spectra of β-carboline-1-carboxylic acid (25) in the negative (NSI-) **(A)** and the positive (NSI+) **(B)** ion mode (upper panel: sample, lower panel: standard).

For component identification, comparison of the MS^1^ and MS^2^ spectra with published ones was extensively used, but for most PQ components no matching published sources could be available. Thus, several standards were first analyzed under the same condition to assist the MS interpretation, including methylnigakinone, nigakinone, and β-carboline-1-carboxylic acid. As shown in Figure 5, the β-carboline-1-carboxylic acid standard (25) was detected in both negative and positive ion mode, with *m/z* 239.0826 and 241.0969, respectively, as precursor ions. The detected active peaks of F125 showed quite similar retention time (tR) and *m/z* compared to the standard both in MS^1^ and MS^2^, indicating that it was identical to the standard, compound 25. Meanwhile, another several active peaks of F47, 88, 108, and 118 (shown in Figure 3) were similarly identified as methylnigakinone (2) and nigakinone (10).

**FIGURE 6:**
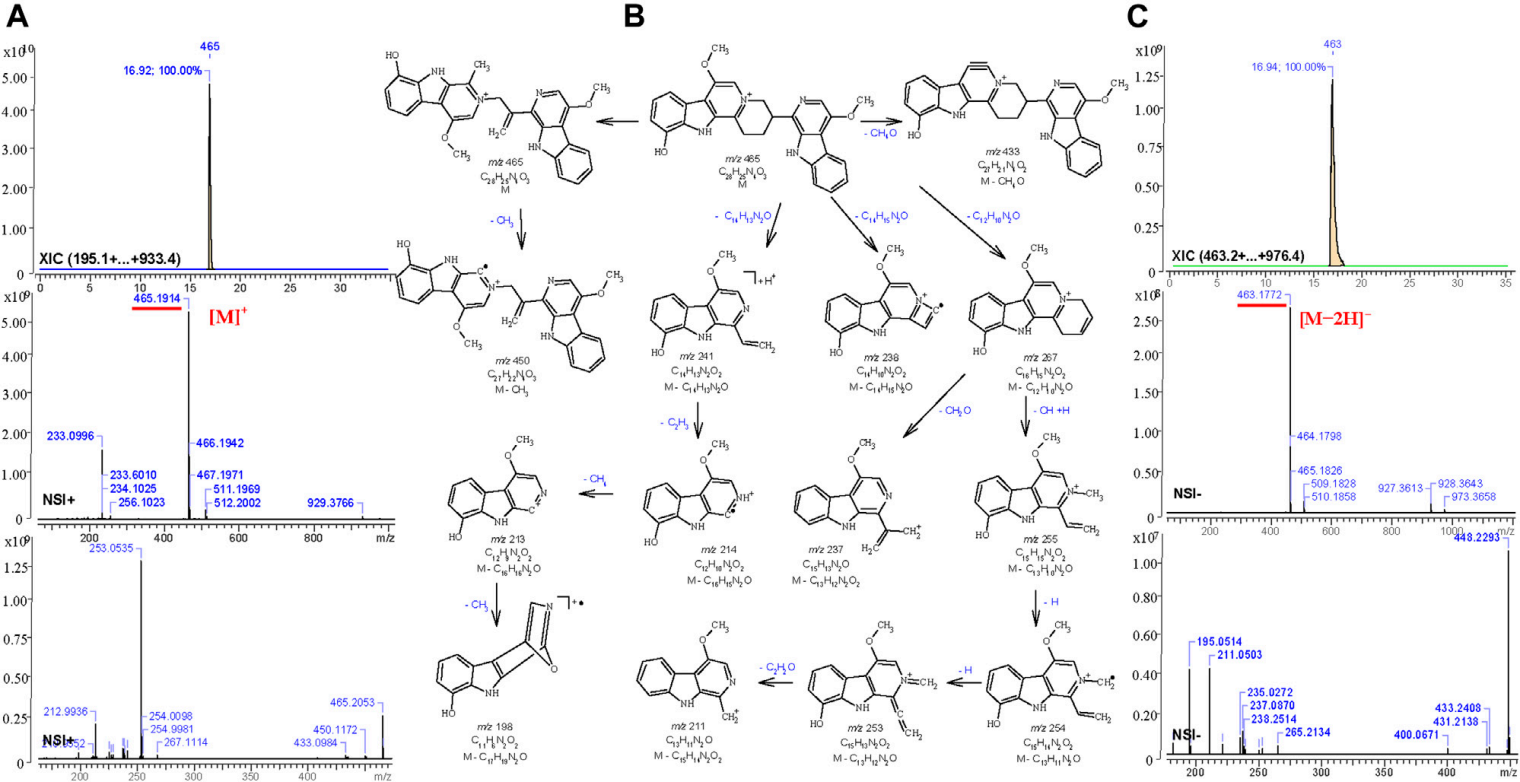
Total ion chromatography analysis (XIC) and MS^1-2^ spectra **(A,C)** of quassidine K **(13)** in the positive (*m*/*z* 465.1914, [C_28_H_25_N_4_O_3_]^+^) and the negative (*m*/*z* 463.1772, [C_28_H_23_N_4_O_3_]^-^) ion mode, and its main mass fragmentation pathway **(B)** in the positive ion mode generated by MS Fragmenter.

For the other compounds where no standards or published spectra were available, the software, MS Fragmenter (Zhou et al., 2014; Zhang Y. et al., 2019) was used to predict and interpret their fragmentation in both positive and negative modes. Taking the fourth active peak in F108 as an example, it was detected in positive and negative ion mode with strong procedures, *m*/*z* 465.1914 [C_28_H_25_N_4_O_3_]^+^ and *m*/*z* 463.1772, [C_28_H_23_N_4_O_3_]^-^,respectively. After searching its structure via Mass Suite Book (ACD/labs) in the COCONUT database (Sorokina et al., 2021) (the biggest publicly available natural product database) and a manually built the PQ database (some components were not included in the COCONUT database), 15 compounds with chemical formula C_28_H_25_N_4_O_3_^+^ or C_28_H_26_N_4_O_3_ were retrieved. Then their mass fragmentation spectra were compared one by one via MS Fragmenter (Tyrkkö et al., 2010), and the best matched one, quassidine K (**13**), was identified as the target, also according to the comparisons of retention time and MS^1^ spectra in positive and negative ion mode. Its fragmentaion in positive ion mode, shown as an example in Figure 6, mainly originates from the cleavage of nitrogen-containing heterocycles. Via the fragmentation analysis, most active peaks of Kumu could be identified and are shown in Table 1.

### Isomers Distiguished by ChromGenius and MS Fragmenter

Although MS Fragmenter can distinguish different fragmentation pattern of compounds, it was still difficult to see the differences for some compounds with quite similar structures. Hence, retention time (tR) calculation can be utilized as a supplementary method to distinguish isomers. Notably, lots of isomers have been reported in PQ, and were also detected in this study. Fraction 60 and 108 showed active peaks with identical *m*/*z* 481.1862 in the positive ion mode, and *m*/*z* 479.1720 and 479.1719 in the negative ion mode. Hence, they were identified as the chemical formula, C_28_H_24_N_4_O_4_ or C_28_H_25_N_4_O_4_^+^. To distiguish their structure, three compounds: kumudine D, picrasidine H, and picrasidine T, were considered after MS Fragmenter analysis as mentioned in the above because they have the same calculated *m*/*z* (481.1870 in positive and 479.1725 in the negative ion mode).

**FIGURE 7:**
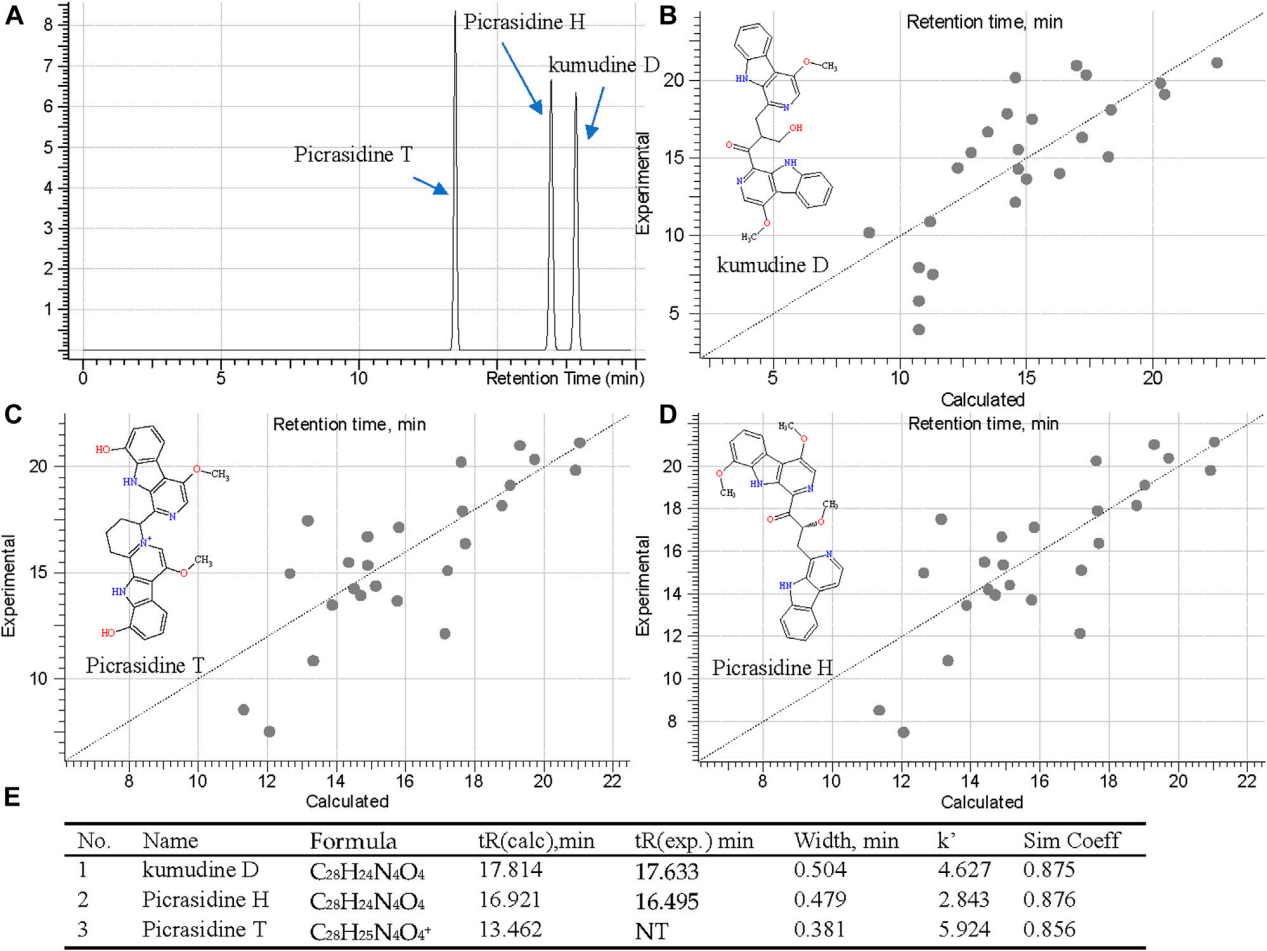
Retention time prediction in chromatography **(A)**, regression curves of three isomers [kumudine D **(B)**, picrasidine T **(C)**, and picrasidine H **(D)**] via ChromGenius and the calculation parameters **(E)**.

Then ChromGenius was utilized for their retention time (tR) calculation, which was performed based on chemical similarity searching (Willett et al., 1998). The calculation was set as the UHPLC-MS/MS condition and showed good prediction according to the main parameters, correction equation (−0.03*tR^2^ + 1.936*tR −6.696) and method coefficient (0.9039). The calculated results are listed in Figure 7E, in which the retention factor k’ was calculated as: k' = (tR – t0)∕t0, where tR stands for retention time, t0 implied dead time, and the similarity coefficient (sim coeff) is calculated based on the hamming distance coefficient (the closer to 1, the better). The predicted tRs of these isomers are shown in Figure 7A, and 25 best-matched points for the calculation are regressed in the curves (Figures 7B–D). Finally, based on the predicted tR, the active peaks in F60 and 108 were identified as kumudine D (9) and picrasidine H (12), respectively.

Four more sets of isomers were similarly analyzed based on their calculated tR: isomers of *m/z* 255.1128 (C_15_H_14_N_2_O_2_) in F60 were picrasidine D (6) and dehydrocrenatidine (7); isomers of *m/z* 479.2078 (C_29_H_27_N_4_O_3_^+^) in F108, 118, and 181 were identified as quassidine L (17), quassidine I (21), and picrasidine F (22), respectively; isomers of *m/z* 509.2183 (C_30_H_29_N_4_O_4_^+^) in F108, 118, and 181 were suggested to be quassidine M (18) and picrasidine S (19), while *m/z* 495.2027 (C_29_H_26_N_4_O_4_) in F108 and 118 were picrasidine C (15) and quassidine J (16), respectively.

To confirm the accuracy of fragmentation and tR calculation for identification, three standards were also subjected to the same calculated method. Taking β-carboline-1-carboxylic acid (25) as an example, the exact masses were 213.0659 ([M+H]^+^) and 211.0513 ([M−H]^−^), and its precursors were detected as 213.0656 and 211.0513 in MS^1^; thus, the chemical formula was calculated as C_12_H_8_N_2_O_2_. Searching in the COCONUT and PQ databases 14 compounds were retrieved. After assigning the MS^2^ fragmentation via MS Fragmenter, three compounds were considered as the best-matching ones, and then, tR calculations were performed to distinguish them. Finally, the compound was identified as β-carboline-1-carboxylic acid. Also, the other two standards could be identified by the same approach. Hence, this can be considered as a method validation for this study.

### The Antimicrobial Spectrum of Three Representative β-carbolines

**FIGURE 8:**
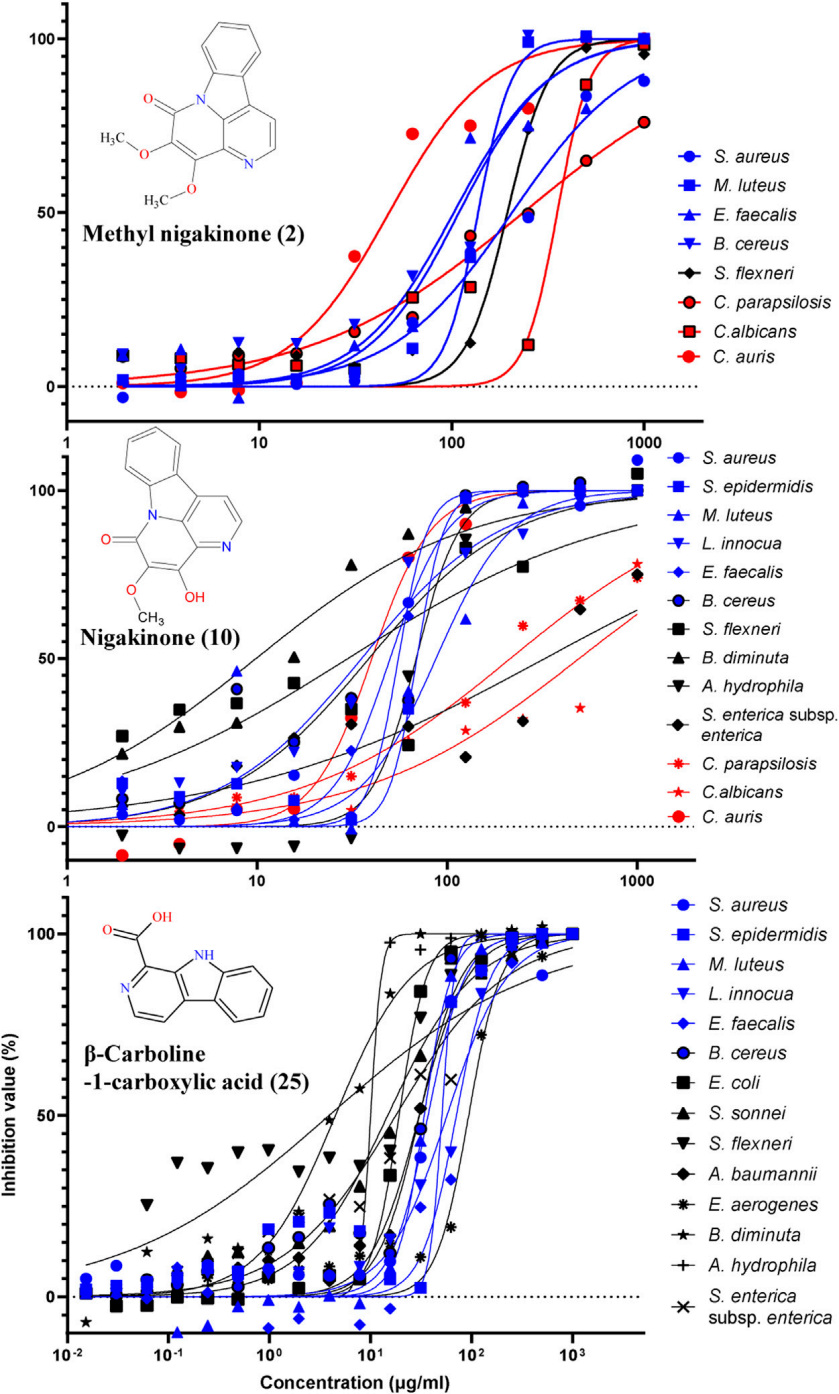
Inhibition of different microorganisms by three β-carbolines from the stem of *Picrasma quassioides*.

The standards of three β-carbolines were also assessed for their IC_50_ (minimum inhibitory concentration to inhibit the growth of 50%) and MBC (minimum bactericidal concentration): methylnigakinone (2), nigakinone (10), and β-carboline-1-carboxylic acid (25). Among them, only methylnigakinone’s antimicrobial activity was reported in a Chinese patent against *Staphylococcus aureus* (MIC = 0.76 mg/ml), *Escherichia coli* (3.09 mg/ml), *Pseudomonas aeruginosa* (3.12 mg/ml), *Streptococcus hemolyticus* (2.46 mg/ml), and *Streptococcus pneumoniae* (1.23 mg/ml) (Wu et al., 2007). As far as we know, our study is the first systematic evaluation of their antimicrobial activities. The tests were done by a 96-well microdilution method against 20 different microorganisms. All three β-carbalines strongly inhibit the growth of many microorganisms and even killed them. Also the GraphPad was used to calculate the IC_50_ while MBC was obtained directly from the agar tests of serial chemical dilution (Table 3, Figure 8).

**TABLE 3:** Antimicrobial activity (μg/ml) of three β-carbolines.

Microbials	Methylnigakinone (2)		Nigakinone (10)		β-Carboline-1-carboxylic acid (25)		Positive control^a^	
	IC50	MBC	IC50	MBC	IC50	MBC	IC_50_	MBC
*S. aureus*	205.70	>500	55.35	>125	47.70	>125	0.28	>125
*S. epidermidis*	NT	NT	69.18	>125	50.88	>125	0.49	125
*M. luteus*	137.10	>250	87.29	>250	33.99	64	2.63	>64
*L. innocua*	NT	NT	35.04	>250	117.80	>125	0.59	>125
*E. faecalis*	109.00	>500	50.07	>125	70.66	>125	9.44^b^	125^b^
*B. cereus*	102.40	>250	38.75	>250	30.48	>125	0.02	>125
*E. coli*	NT	NT	NT	NT	19.17	>125	0.02	<3.91
*P. aeruginosa*	NT	NT	NT	NT	NT	NT	0.02	7.81
*S. sonnei*	NT	NT	NT	NT	14.81	>125	0.02	31.25
*S. flexneri*	194.80	>250	29.99	>250	3.96	125	0.02	<3.91
*A. baumannii*	NT	NT	NT	NT	30.28	>125	0.17	15.62
*E. aerogenes*	NT	NT	NT	NT	93.65	>125	0.04	>125
*B. diminuta*	NT	NT	10.46	>64	4.50	64	2.26	>64
*A. hydrophila*	NT	NT	68.64	>64	10.03	64	<0.01	0.02
*S. enterica* subsp. *enterica*	NT	NT	490.12	>500	19.34	>64	0.01	2.00
*C. parapsilosis*	236.00	>500	201.50	>500	NT	NT	0.13^c^	12.56^c^
*C.albicans*	356.90	>500	493.80	>500	NT	NT	0.01^c^	12.56^c^
*C. auris*	32.82	>125	31.91	>125	NT	NT	0.10^c^	>50^c^
*C. glabrata*	NT	NT	NT	NT	NT	NT	0.12^c^	>12.56^c^
*S. cerevisiae*	NT	NT	NT	NT	NT	NT	0.44^c^	>50^c^

a Positive control: Ciprofloxacin.

b Chloramphenicol.

c Miconazole.

From the result of antimicrobial test, compound 2 (C2) can inhibit four Gram-positive, one Gram-negative bacteria, and three fungi; however, most of the IC_50_ values were above 100 μg/ml. C10 was active against more microorganisms, including six G^+^, four G^–^ bacteria, and three fungi, and showed mostly somewhat higher activity than C2. C10 differs from C2 by a methylation at the 4’-hydroxy position. The methylation can reduce the chemical sensitivity, which was one of mechanism of antimicrobial resistance (Malone and Gordon, 2016; Warrier et al., 2016), while other study also indicated the methylation can also improve antimicrobial potency and broaden the spectrum (Benhamou et al., 2015), indicating that methylation worked differently for diverse compounds. According to the IC_50_ and MBC results, it was clear that the methylation reduced the antibacterial, but not the antifungal activity of C10, suggesting its different mechanism of action on bacteria vs. fungi.

An interesting result was obtained for *Candida auris*, an important emerging fungal pathogen, which was first described in 2009 and has spread across six continents as a cause of infections in hospitals (Meis and Chowdhary, 2018; Proctor et al., 2021). Both C2 and C10 were found to strongly suppress the growth of *C. auris* with a similar IC_50_ (around 30 μg/ml). Furthermore, C25 exhibited higher antibacterial activity and broader spectrum than both C2 and C10, including six G^+^ and eight G^–^ bacteria, but it showed no antifungal activity. Notably, most G^–^ bacteria, such as *E. coli*, *S. sonnei*, *S. flexneri*, *B. diminuta*, *A. hydrophila*, and *S. enterica* subsp. *enterica* were sensitive for C25 (with IC_50_ mostly below 20 μg/ml). Therefore, all three compounds were considered as having potential for antibiotic development.

### Bioactivity Discussion of Kumu Compounds

To better comparing the bioactivities of the chemicals detected in this study, all the related literatures were checked, especially for their antimicrobial activity. 1-acetyl-β-carboline (1)(OHMOTO and KOIKE, 1982), methylnigakinone (2)(Jiang and Zhou, 2008), and melianone (3) were identified from fraction 47, where C1 was already reported as active against several laboratory and clinical strains (Savidge and Sorg, 2019)(Chen et al., 2018), such as *S. aureus,* with IC_50_ below 260 μg/ml (Shin et al., 2010). C2 has shown antiulcer effects (Omoja et al., 2014), significant cytotoxicity against CNE2 and HepG2 cancer cells (Jiang and Zhou, 2008; Mohd Jamil et al., 2020), and antiviral activity against Coxsackie virus B3 (with an IC_50_ as 7.4 μM (Zhang et al., 2013)), and its antimicrobial activity was documented and patented (Mitscher et al., 1975; Wu et al., 2007). Moreover, C3 (Zhao et al., 2019) was active against *Salmonella* ser. typhi with both MIC and MBC of 0.053 µM (Veni et al., 2020) against *S. aureus* with agar diffusion inhibition zone of 9 mm (Biavatti et al., 2001), and it was also documented for anticancer, antivirus, anti-gout, anti-depressant, and antifeeding activities (Su et al., 1990; Ishaq, 2016; Liu Y. et al., 2019; Zhao et al., 2019; Anita and Praveena, 2020; Sundar and Aarthy, 2020).

In fraction 60, six active chemicals were identified: 1-methoxycarbonyl-β-carboline (4), dehydrocrenatine (5), picrasidine D (6), dehydrocrenatidine (7), canthin-6-one (8), and kumudine D (9). Compound 4 can inhibit tobacco mosaic virus (TMV) replication with EC_50_ of 0.179 mM (Chen et al., 2009b) and had significant inhibitory activity on various plant pathogenic bacteria and fungi (Gao et al., 2020). C5 was reported for antimalarial, anti-inflammatory, antiparasitic, and antiprotozoal activities (Kita et al., 2004; Van Baelen et al., 2009; Liu P. et al., 2019; Saiin et al., 2019), but ours is the first report of its antibacterial activity to the best of our knowledge. C6 and C7 were reported for their cytotoxicity, anti-inflammatory, and antivirus activities (Ohmoto and Koike, 1988; Han et al., 2001; Chen et al., 2009b; Lee et al., 2009; Liu P. et al., 2019); their antibacterial activity is reported here for the first time. C8 showed anticancer, antimalarial, anti-​inflammatory, antidiabetic, anti-plasmodium, antiprotozoal, antibacterial, antifungal, and antivirus activities (Lagoutte et al., 2008; Ross et al., 2008; Almeida et al., 2011; Arias et al., 2011; Cebrian-Torrejon et al., 2011; Agrawal et al., 2013; Rashed et al., 2013; Amanulla et al., 2017; Cho et al., 2018; Makong et al., 2019); it was active against *S. aureus* (of inhibition zone diameter of 6.1 mm), but no activity against *E. coli* was observed (Casciaro et al., 2019), which is the same as our result. C9 showed cytotoxicity against HepG2 and 3B cells, but there are no reports thus far about its antimicrobial activity.

Fraction 88 only showed one active peak, identified as nigakinone (10), which has documented cytotoxicity, antivirus, antimalarial, and anti-inflammatory activities (Kuo et al., 2003; Chen et al., 2009b; Liu P. et al., 2019). Both fraction 108 and 118 had 10 active peaks, as shown in Figure 3. The active peaks in F108 were identified as nigakinone (10), picrasidine A (11), picrasidine H (12), (±)-quassidine K (13), kumudine B (14), picrasidine C (15), quassidine J (16), quassidine L (17), quassidine M (18), and picrasidine S (19). Many similar compounds were found in F118, including C10, 13, 16, 17, 18, and 19, in addition to four other compounds: picrasidine G (20), quassidine I (21), picrasidine F (22), and quassidine A (23). It can be expected that the composition of compounds eluting from successive steps in a gradient may overlap, especially if the steps are small. For their bioactivities, C11 was reported as a TMV inhibitor (Chen et al., 2009a) and C12 (KOIKE and OHMOTO, 1986) showed none of anti-inflammatory effects and cytotoxicity against RAW 264.7 cells (Liu P. et al., 2019), while C13 had good cytotoxicity against the human cervical HeLa cell line, with an IC_50_ value around 20 μM (Guo et al., 2020). C14 has anti-hepatoma potential l (Zhao et al., 2019d), and C15 exhibited anti-inflammatory activity (Liu P. et al., 2019) and cytotoxicity against RAW 264.7 (Liu P. et al., 2019), esophageal squamous carcinoma cells (Shi et al., 2018), and also beneficial effects in cerebral protection (Sasaki et al., 2016). From the literatures, C16, C19, C20, and C21 had cytotoxicity against human cervical HeLa, gastric MKN-28 cancer, and mouse melanoma B-16 cancer cells (Jiao et al., 2015), and C21 also strongly inhibited osteoclast formation (Kong et al., 2017). C17 exerted beneficial effects in the cerebral protection (Sasaki et al., 2016), and the trifluoroacetate of both C17 and C18 showed potential anti-Alzheimer’s disease activity (Zhang et al., 2020). C20 had antiprotozoal activity (Kita et al., 2004) and was also active against RAW 264.7 (Liu P. et al., 2019) and triple-negative breast cancer cells (MDA-MB 468) (Yamashita et al., 2017). C22 was toxic against HeLa cells and active against several methicillin-sensitive and -resistant strains of *S. aureus* (MRSA), while C19 and C20 were also reported for their anti-MRSA activity (Guo-hua et al., 2015). For C23, only its anti-inflammatory activity was studied (Jiao et al., 2010b).

Only two active compounds were found in F125: picrasidine I (24) and β-carboline-1-carboxylic acid (25). C24 was known to have a strong antiosteoclastogenic effect (Kong et al., 2017) but no other effects were reported thus far. For C25, the previous bioactivity studies implied it can inhibit the growth of K-562 and SGC-7901 cell lines (Lai et al., 2014) and had potential for attenuating bleomycin-induced pulmonary fibrosis (Cui et al., 2019), while none anti-inflammatory activity (Liu P. et al., 2019) and none photocytotoxicity against lung (A549) and colon (Colon-26) cancer cells (Reddy et al., 2019).

To summarize, 18 compounds identified in our study are first reported here for their activity against *S. aureus* or *E. coli*, including 1-methoxycarbonyl-β-carboline (4), dehydrocrenatine (5), picrasidine D (6), dehydrocrenatidine (7), kumudine D (9), nigakinone (10), picrasidine A (11), picrasidine H (12), (±)-quassidine K (13), kumudine B (14), picrasidine C (15), quassidine J (16), quassidine L (17), quassidine M (18), quassidine I (21), quassidine A (23), Picrasidine I (24), and β-carboline-1-carboxylic acid (25). Notably, 24 active compounds were β-carbolines or dimers thereof. This class is a promising scaffold for the design and development of antibacterial compounds. It is worth to find lots of related bioactive compounds in plant extracts, since the biosynthesis of secondary metabolites often generates significant amounts of intermediates of the biosynthetic pathway, some of which may share bioactivities. The number of bioactive compounds identified from a single plant is large in this study, although it cannot be sure that all the identified β-carbolines originate from a single biosynthetic pathway here, which could be helpful and pending for future studies.

## Conclusion

In summary of this study, the interpretation method using two types of columns for chemical isolation and UHPLC-Orbitrap- Ion-Trap Mass Spectrometer for chemical identification was developed. The standard comparison, database searching, MS Fragmenter, and ChromGenius were introduced for the structure analysis. The results showed that high-resolution mass spectrometry combined with mass fragmentation and retention time calculation was a powerful tool to identify natural products, especially for distinguishing isomers with similar structures. A total of 25 active compounds were isolated and identified from Kumu against *S. aureus* or *E. coli*, including five types of isomers, in which 18 components’ antimicrobial activities were firstly reported in this study. Notably, 24 active chemicals belonged to β-carboline or its dimer, which class exhibited as a promising scaffold for the design and development of potent and selective antibacterial compounds. The antimicrobial components reported here provide a basis for the medicinal usage of themselves and Kumu, and are beneficial for the scientific development of quality standards and its therapeutic utilization, indicating that the method can be used to figure out active chemical basis for other complex materials.

## Data Availability

The raw data supporting the conclusions of this article will be made available by the authors, without undue reservation.
