# Corrigendum: Bioassay-Guided Interpretation of Antimicrobial Compounds in Kumu, a TCM Preparation From *Picrasma quassioides*’ Stem via UHPLC-Orbitrap-Ion Trap Mass Spectrometer Combined With Fragmentation and Retention Time Calculation

**DOI:** 10.3389/fphar.2022.897243

**Published:** 2022-04-25

**Authors:** Haibo Hu, Changling Hu, Jinnian Peng, Alokesh Kumar Ghosh, Ajmal Khan, Dan Sun, Walter Luyten

**Affiliations:** ^1^ Department of Biology, Animal Physiology and Neurobiology Section, KU Leuven, Leuven, Belgium; ^2^ National Engineering Research Center for Modernization of Traditional Chinese Medicine—Hakka Medical Resources Branch, School of Pharmacy, Gannan Medical University, Ganzhou, China; ^3^ Laboratory for Functional Foods and Human Health, Center for Excellence in Postharvest Technologies, North Carolina Agricultural and Technical State University, North Carolina Research Campus, Kannapolis, NC, United States; ^4^ College of Life Sciences, NanKai University, Tianjin, China

**Keywords:** *Picrasma quassioides*, kumu, beta-carboline, orbitrap elite, MS fragmenter, fragmentation prediction

In the original article, there was a mistake in Table 3 as published. The IC_50_, and MBC calculations of positive controls were unintentionally incorrect when generated by software Graphpad, and may make people confused about their high IC_50_ values in the original manuscript. Hence, the tests of these compounds were repeated twice again to confirm these results. The corrected Table 3 appears below.

**TABLE 3:** Antimicrobial activity (μg/ml) of three β-carbolines.

Microbials	Methylnigakinone (2)		Nigakinone (10)		β-Carboline-1-carboxylic acid (25)		Positive control^a^	
	IC50	MBC	IC50	MBC	IC50	MBC	IC_50_	MBC
*S. aureus*	205.70	>500	55.35	>125	47.70	>125	0.28	>125
*S. epidermidis*	NT	NT	69.18	>125	50.88	>125	0.49	125
*M. luteus*	137.10	>250	87.29	>250	33.99	64	2.63	>64
*L. innocua*	NT	NT	35.04	>250	117.80	>125	0.59	>125
*E. faecalis*	109.00	>500	50.07	>125	70.66	>125	9.44^b^	125^b^
*B. cereus*	102.40	>250	38.75	>250	30.48	>125	0.02	>125
*E. coli*	NT	NT	NT	NT	19.17	>125	0.02	<3.91
*P. aeruginosa*	NT	NT	NT	NT	NT	NT	0.02	7.81
*S. sonnei*	NT	NT	NT	NT	14.81	>125	0.02	31.25
*S. flexneri*	194.80	>250	29.99	>250	3.96	125	0.02	<3.91
*A. baumannii*	NT	NT	NT	NT	30.28	>125	0.17	15.62
*E. aerogenes*	NT	NT	NT	NT	93.65	>125	0.04	>125
*B. diminuta*	NT	NT	10.46	>64	4.50	64	2.26	>64
*A. hydrophila*	NT	NT	68.64	>64	10.03	64	<0.01	0.02
*S. enterica* subsp. *enterica*	NT	NT	490.12	>500	19.34	>64	0.01	2.00
*C. parapsilosis*	236.00	>500	201.50	>500	NT	NT	0.13^c^	12.56^c^
*C.albicans*	356.90	>500	493.80	>500	NT	NT	0.01^c^	12.56^c^
*C. auris*	32.82	>125	31.91	>125	NT	NT	0.10^c^	>50^c^
*C. glabrata*	NT	NT	NT	NT	NT	NT	0.12^c^	>12.56^c^
*S. cerevisiae*	NT	NT	NT	NT	NT	NT	0.44^c^	>50^c^

a Positive control: Ciprofloxacin.

b Chloramphenicol.

c Miconazole.

The authors apologize for this error and state that this does not change the scientific conclusions of the article in any way. The original article has been updated.

